# *Haemophilus parasuis* Infection Disrupts Adherens Junctions and Initializes EMT Dependent on Canonical Wnt/β-Catenin Signaling Pathway

**DOI:** 10.3389/fcimb.2018.00324

**Published:** 2018-09-12

**Authors:** Kexin Hua, Yangjie Li, Hufeng Zhou, Xueying Hu, Yushan Chen, Rongrong He, Rui Luo, Rui Zhou, Dingren Bi, Hui Jin

**Affiliations:** ^1^State Key Laboratory of Agricultural Microbiology, Huazhong Agricultural University, Wuhan, China; ^2^College of Animal Medicine, Huazhong Agricultural University, Wuhan, China; ^3^Hubei Provincial Key Laboratory of Preventive Veterinary Medicine, Huazhong Agricultural University, Wuhan, China; ^4^Department of Medicine, Brigham and Women's Hospital, Boston, MA, United States; ^5^Department of Immunology and Microbiology, Harvard Medical School, Boston, MA, United States; ^6^Brain and Cognition Research Institute, Wuhan University of Science and Technology, Wuhan, China; ^7^Key Laboratory of Occupational Hazard Identification and Control in Hubei Province, Wuhan University of Science and Technology, Wuhan, China

**Keywords:** *Haemophilus parasuis*, Wnt/β-catenin, E-cadherin, adherens junctions, EMT

## Abstract

In this study, animal experimentation verified that the canonical Wnt/β-catenin signaling pathway was activated under a reduced activity of p-β-catenin (Ser33/37/Thr41) and an increased accumulation of β-catenin in the lungs and kidneys of pigs infected with a highly virulent strain of *H. parasuis*. In PK-15 and NPTr cells, it was also confirmed that infection with a high-virulence strain of *H. parasuis* induced cytoplasmic accumulation and nuclear translocation of β-catenin. *H. parasuis* infection caused a sharp degradation of E-cadherin and an increase of the epithelial cell monolayer permeability, as well as a broken interaction between β-catenin and E-cadherin dependent on Wnt/β-catenin signaling pathway. Moreover, Wnt/β-catenin signaling pathway also contributed to the initiation of epithelial-mesenchymal transition (EMT) during high-virulence strain of *H. parasuis* infection with expression changes of epithelial/mesenchymal markers, increased migratory capabilities as well as the morphologically spindle-like switch in PK-15 and NPTr cells. Therefore, we originally speculated that *H. parasuis* infection activates the canonical Wnt/β-catenin signaling pathway leading to a disruption of the epithelial barrier, altering cell structure and increasing cell migration, which results in severe acute systemic infection characterized by fibrinous polyserositis during *H. parasuis* infection.

## Introduction

*Haemophilus parasuis* is the etiological agent of Glässer's disease, an important bacterial disease in swine worldwide, which causes serious economic loss to the global pig industry (Olvera et al., 2007; Frandoloso et al., 2012). The sudden death caused by *H. parasuis* is a typical acute systemic inflammation with massive fibrin exudates in the pleuroperitoneal cavity as well as membranes (Nedbalcova et al., 2006). Therefore, deeper insight into the role of *H. parasuis*-induced disruption of epithelial cellular junctions may open new venues for controlling acute infectious diseases. Here, we selected two epithelial cells as models, kidney-15 (PK-15) and Newborn pig tracheal (NPTr) cells. Previous studies demonstrate that PK-15 cells are suitable models for studying *H. parasuis*-induced inflammation and immunity (Chen et al., 2012, 2015a,b). In addition, NPTr cells are involved in the initial colonization of *H. parasuis* (Bouchet et al., 2009), and are therefore suitable for exploring its pathogenesis.

Epithelial cells form the important junction between the external environment and internal organs, and act as a barrier to prevent various external adverse factors such as microorganisms gaining access into the body and blood (Nagafuchi, 2001; Moens and Veldhoen, 2012). It is well known that in epithelial tissues cell junctions are especially abundant (Jefferson et al., 2004), among which AJs mainly maintain organization structure (Turner, 2009). The major component of AJs is the transmembrane protein E-cadherin, which establishes Ca^2+^ dependent-interaction with E-cadherin molecules of neighboring cells (Du et al., 2014). The cytoplasmic domain of E-cadherin connects with the catenin protein family including α- and β-catenin, and this complex is associated with the actin cytoskeleton to form stable AJs (Nagafuchi, 2001). The E-cadherin/β-catenin complex maintains the integrity of epithelial cell-cell contact as well as epithelial morphology, and keeps Wnt/β-catenin signals in check (Tian et al., 2011). β-catenin is a multifunctional protein in the Wnt signaling pathway and has at least two different roles (O'Connor et al., 2011): when not stimulated by Wnt signals, cytoplasmic β-catenin mainly connects with the proximal C-terminal domain of E-cadherin in cell membranes as an integral part of AJs; Upon Wnt stimulation, accumulated β-catenin then translocates into the nucleus to combine with TCF/LEF (T cell factor/lymphoid enhancer-binding factor) transcription factors (Clevers and Nusse, 2012).

β-Catenin in the nucleus initiates transcription regulation of target genes such as matrix metalloproteinase7 (MMP7), cyclooxygenase2 (COX2), and plasminogen activator inhibitor-1 (PAI-1), which are involved in cell apoptosis, proliferation, carcinogenic effects, cell migration, cell adhesion (Brabletz et al., 1999; Pu et al., 2007; He et al., 2010) and epithelial–mesenchymal transition (EMT) (Adhim et al., 2011; Omori et al., 2016; Zhang et al., 2017). EMT is characterized by the loss of uniform cell shape, apical-basal polarity as well as strong cell-cell adherent junctions, and the gain of mesenchymal attributes including front-back polarity and loose cell attachment (Zhang et al., 2016). It has been associated with pathological processes of fibrosis and cancer progression requiring epithelial cell migration (Lamouille et al., 2014), and responsible for tight junctions by gaining mesenchymal markers such as N-Cadherin, Vimentin, Fibronectin, and TG2-Snail-E-cadherin axis (Rout-Pitt et al., 2018). The loss of E-cadherin expression, regulated by Wnt signaling, is considered to be a fundamental event in EMT (Thiery et al., 2009). Also, in the nucleus of Wnt activated cells, together with TCF/LEF, β-catenin binds to the promoter region of the key transcription factors (e.g., SNAIL1) to initialize and maintain the process of EMT by directly regulating EMT target genes, which is a key step in development, wound healing, and cancer development (Zhang et al., 2016).

In this study, we explored the hypothesis that *H. parasuis* infection disrupts AJs and initializes the process of EMT dependent on the canonical Wnt/β-catenin signaling pathway in epithelial cells. Our study provides a previously unexplored perspective of canonical Wnt/β-catenin signaling in *H. parasuis* infection and suggests a new pathological angle for understanding acute inflammation.

## Materials and methods

### Animals and tissues collection

Nine 30-day-old piglets were randomly divided into three treatment groups equally. Three groups of piglets were injected intraperitoneally with 1 ml normal saline as control, 2 × 10^9^ colony CFU of avirulent *H. parasuis* strain HN0001 and 2 × 10^9^ colony CFU of virulent *H. parasuis* strain SH0165 respectively, and for strain suspension preparation, 1 mL sterile normal saline was used as diluent. The piglets were treated identically with. *H. parasuis* SH0165 group piglets showed significant clinical symptoms on 24 h post-inoculation and all piglets were euthanized 24 h after injection. One portion of lungs and kidneys including medulla and cortex was fixed in 4% paraformaldehyde and then used for immunohistochemistry, and another was snap frozen in liquid nitrogen and then stored at −80°C until protein extraction. The experiments were carried out in strict according to the Guide for the Care and Use of Animals in Research of the People's Republic of China. All experimental procedures were approved by The Scientific Ethic Committee of Huazhong Agricultural University (HZAUSW-2015-013).

### Immunohistochemistry

Samples from lungs and kidneys were embedded with paraffin after fixed in paraformaldehyde overnight, and then tissues were cut at the thickness of 4 μm to perform immunohistochemistry. The sections were deparaffinized, rehydrated and treated with 3% H_2_O_2_ (Sigma-Aldrich). After repairing antigen with microwave for 30 min, the sections were blocked with 5% bovine serum albumin (Sigma-Aldrich) for 20 min and incubated with primary antibody anti-β-catenin (at a dilution of 1:100) (Cell Signaling Technology, USA) at room temperature for 2 h. After washing 3 times with PBS (pH7.4), the sections were incubated with secondary antibody for 30 min and then treated with Strept Avidin-Biotin Complex (Boster Technology, China) and 3,3′-diaminobenzidine (Boster Technology, China) after washing with water. The nucleus was counterstained with hematoxylin for 30 s. The results were carried out with Nikon 80i microscope (Nikon, Tokyo, Japan).

### Cell and bacterial culture

PK-15 cell line was obtained from ATCC (CCL-33) and new-born pig trachea (NPTr) cells were separated from piglets refer to the method of Ferrari (Ferrari et al., 2003). Both cell lines were cultured in DMEM (Gibco) supplemented with 10% FBS (Gibco) at 37°C and 5% CO_2_ without any antibiotics. *H. parasuis* SH0165 strain (serovar 5, high virulent strain) and HN0001 strain (serovar 6, non-virulent strain) were cultured in tryptic soy broth (TSB; Difco Laboratories, USA) supplemented with 10% newborn calf serum (NBCS) (Gibco) and 0.2 mg/ml nicotinamide adenine dinucleotide (NAD) (Bio-sharp, China) without any antibiotics. After incubation at 37°C with circular agitation (200 rpm) overnight, bacteria were harvested by centrifugation at 5,000 × g for 5 min, washed with PBS for three times and resuspended in fresh DMEM medium to infect cells *in vitro*. Moreover, we have tested the numbers of bacteria at 6 h, 12 h, 24 h after adding into cells, there was no significant difference in quantity of bacteria whether the inhibitors were added into medium (Supplemental Figure 1).

### Plasmids, antibodies and reagents

TOPflash luciferase reporter plasmid with Wild type TCF/LEF binding sites and FOPflash luciferase reporter plasmid with mutated TCF/LEF binding sites were obtained from Upstate biotechnology (USA). Monoclonal antibodies against E-cadherin (sc-8426) and polyclonal antibodies against β-catenin (sc-7199) were purchased from Santa Cruz Biotechnology (USA). Polyclonal antibodies against phospho-β-catenin (Ser33/37/Thr41) (#9561) and monoclonal antibodies against Caveolin-1 (#3267), PCNA (#2586) and Vimentin (#5741) were purchased from Cell Signaling Technology (USA). Monoclonal antibodies β-actin (#AA128) and GAPDH (AF1186) were obtained from Beyotime (China). Lithium chloride (LiCl) (L7026) was obtained from Sigma-Aldrich (USA). TGF-β1 (100-21-2) was obtained from Peprotech (USA). Alexa Fluor 488-labeled Goat Anti-Rabbit IgG(H+L) (A0423) and Alexa Fluor 555-labeled Donkey Anti-Mouse IgG(H+L) (A0460) were purchased from Beyotime (China). Wnt/β-catenin inhibitor ICG001 (S2662), IWR-1-endo (S7086) were obtained from Selleck (USA). IWR-1-endo inhibits Wnt signaling by inhibiting Wnt-induced accumulation of β-catenin and ICG001 antagonizes Wnt/β-catenin/TCF-mediated transcription and specifically binds to CREB-binding protein (CBP) (Chen et al., 2009; Lazarova et al., 2013). Each type of inhibitor was added into cells to the final concentration of 10 μM for 2 h before stimulation with *H. parasuis* or LiCl.

### Plasmid transfection and luciferase assay

To assess the activation of Wnt/β-catenin pathway, cells seeded in 96 well plates were transfected with 0.1 μg/well of either TOPflash or FOPflash and 0.1 μg/well of pRL-TK by 0.5 μg/well Lipofectamine 2000 (Invitrogen, USA) following the manufacturer's instructions. At 24 h after transfection, cells were treated or untreated with various inhibitors for 2 h before stimulation with *H. parasuis* or LiCl. Cells were lysed 24 h after stimulation and the relative firefly and renilla luciferase activity were detected with a dual-luciferase reporter assay system (Promega, USA) following the manufacturer's instructions.

### Western blotting

Total protein of 0.1 g tissue samples of each tissue was extracted by 1 mL RIPA Lysis Buffer (P0013B, Beyotime, China) supplemented with 1 mM protease inhibitor cocktail (Roche). Protein concentration was calculated by BCA assays (Pierce, Rockford, IL, USA) following the manufacturer's instructions and 15 μg protein of each sample was used for western blot analysis.

PK-15 cells or NPTr cells were seeded in 6-well plates and cultured until 70–80% confluence, and then were treated or untreated with pathway inhibitors for 2 h before stimulated with *H. parasuis* or LiCl. Cells were harvested at the indicated time points by 200 μL/well Cell lysis buffer for Western and IP (P0013, Beyotime, China) supplemented with 1 mM protease inhibitor cocktail (Roche). The subcellular fractionation of proteomic samples from membrane, cytoplasm, and nucleus was obtained by Qproteome cell compartment kit (Qiagen) following the manufacturer's instructions. Western blot analysis was performed as described previously (Chen et al., 2012).

### Indirect immunofluorescence

For all indirect immunofluorescence analysis, cells were seeded on 13-mm sterilized coverslips and cultured overnight to 50–60% confluence. Cells were treated or untreated with indicated inhibitors for 2 h, and then stimulated with *H. parasuis*, LiCl or TGF-β1. Twelve hours after stimulation, cells were washed with phosphate buffered-saline (PBS, Gibco) for 3 times and fixed with 4% paraformaldehyde (Sigma) for 15 min. The cells were then permeabilized with 0.1% Triton X-100 (Sigma) for 15 min followed by blocking with 5% bovine serum albumin (Sigma-Aldrich) for 1 h at room temperature. After blocking, cells were incubated with primary antibody for 2 h and then incubated with AlexaFluor-conjugated secondary antibodies for 1 h after washed by PBS for 3 times. Prior to imaging, nucleus is counterstained with 4′-6-diamidino-2-phenylindole (DAPI) (Invitrogen). Coverslips were imaged at 40× by a Zeiss LSM 510 Meta confocal microscope (Carl Zeiss, Germany).

### TEER measurement

PK-15 or NPTr cells were seeded on upper compartment of transwell cell culture system (0.33 cm^2^ of surface area and 0.4 μm of pore size, Costar, USA) and cultured for 48 h to form confluent monolayers with a resistance of 300 Ω^*^cm^2^. Cells were subsequently treated or untreated with indicated inhibitors (10 μM) for 2 h, and then stimulated or unstimulated with *H. parasuis* (10^8^ CFU/mL) or LiCl (20 mM). At 0, 6, 12, 18, and 24 h post-infection, the EVOM2 volt ohm meter (World Precision Instruments, Sarasota, FL) was used to measure the transepithelial electrical resistance (TEER) of cell monolayers according to the manufacturer's recommendations. Three independent experiments were performed.

### RNA extraction and quantitative real-time RT-PCR (qRT-PCR) analysis

Total RNA was isolated from cells by TRIzol reagent (Invitrogen, USA) at the indicated periods of time post-infection, 1 μg RNA of each sample was reverse transcribed into cDNA with iScript cDNA synthesis kit (Bio-Rad) and the quantitative real time PCR reactions were performed by SYBR Green Supermix (Bio-Rad) following the manufacturer's instructions. Transcripts of three individual samples were normalized to the corresponding porcine glyceraldehyde-3-phosphate dehydrogenase (GAPDH) mRNA level. The sequences of the primers are shown in Table 1.

**Table 1:** qPCR primers used in this study.

**Primer**	**Sequence (5′-3′)**
E-cadherin-F	GCACCAACCCTCCTGAGTGT
E-cadherin-R	ACACCACTTTGCCATCCA
Collagen IV-F	ACGGCTACTCTCTGCTCTAC
Collagen IV-R	GAAGTTGCAGACGTTGTTG
Cytokeratin-F	GGCGAGGACTTCAATCTTG
Cytokeratin-R	ACACCACTTTGCCATCCA
N-cadherin-F	CACAGATATGGAAGGCAATC
N-cadherin-R	GGCAGTAAACTCTGGAGGAT
Snail-F	CGCTCTTTTCTCGTCAGGAA
Snail-R	CATAGGGCTGCTGAAAAGTG
Vimentin-F	GGAGTGGTACAAGTCCAAGT
Vimentin-R	TCTCCGGTACTCATTTGAC
S100A4-F	GAGAAGGCCCTCGATGTGAT
S100A4-R	TCAGCAACTCCTTTAGCTCAGA
MMP7-F	GGCCGTACTGTGTGCTGTGT
MMP7-R	CCTTTGTTTTTGAGTTGGATGGA
COX2-F	GAAGCGCTCTATGGTGACATTG
COX2-R	GCATCTGGGCGAGGCTTT
PAI-1-F	TGCCGCCCCCTACGA
PAI-1-R	TGAGCTGAGCGTCCAGAATG
GAPDH-F	CCCCAACGTGTCGGTTGT
GAPDH-R	CCTGCTTCACCACCTTCTTGA

### Morphological observation and wound healing assay

PK-15 or NPTr cells were cultured in 60-mm plates to 90% confluence. The morphological changes of cells were photographed with an inverted microscope (Olympus IX73, Japan) after stimulated by *SH0165* (10^8^ CFU/mL) or TGF-β1 (10 ng/mL) for 12 h. Wnt/β-catenin inhibitor ICG001 was added 2 h before infection. For the wound healing assay, cells were seeded in 6 well plates and cultured to 90% confluence, and then the monolayer of cells was wounded with sterile pipette tips. The images were observed with an inverted microscope (Olympus IX73, Japan) after treatment described above. Each experiment was performed at least three times independently.

### Knockdown of MMP7, COX2 and PAI-1 with siRNA

Sequence-specific small interfering RNAs (siRNAs) of three genes and siNegative oligonucleotide (negative control) were purchased from GenePharma (China). Cells seeded at 24 well plates were cultured to 70% confluence and transfected with 15 pmol/well sequence-specific siRNAs or siNegative by 1.5 μg/well lipofectamine 2000 (Invitrogen). Twenty-Four hours after transfection, cells were collected to perform qRT-PCR as described above. The primers used in qRT-PCR were presented in Table 1 and the sequences of siRNAs are shown in Table 2.

**Table 2:** The sequences of siRNAs used in the study.

**siRNA**	**Sequences (5′to 3′)**
PAI-1-1	5′ CCCUCCGUCAACUGUACAATT 3′ 5′ UUGUACAGUUGACGGAGGGTT 3′
PAI-1-2	5′ CCUCUUCCACAAGUCUGAUTT 3′ 5′ AUCAGACUUGUGGAAGAGGTT 3′
PAI-1-3	5′ CCUCCUCUACGGCCAUUAUTT 3′ 5′ AUAAUGGCCGUAGAGGAGGTT 3′
MMP7-1	5′ GGAUGCUAAUAGUUUGGAATT 3′ 5′ UUCCAAACUAUUAGCAUCCTT 3′
MMP7-2	5′ CCCAUUGAGCUUUAAGAAATT 3′ 5′UUUCUUAAAGCUCAAUGGGTT3′
MMP7-3	5′ CCAACCUACGGAGAACUAUTT 3′ 5′ AUAGUUCUCCGUAGGUUGGTT 3′
COX2-1	5′ GCACCCGAACAGGAUUCUATT 3′ 5′ UAGAAUCCUGUUCGGGUGCTT 3′
COX2-2	5′ GGGCACAAACAUGAUGUUUTT 3′ 5′ AAACAUCAUGUUUGUGCCCTT 3′
COX2-3	5′ GCCCUAUCGAUCAUUUGAATT 3′ 5′ UUCAAAUGAUCGAUAGGGCTT 3′

### Statistical analysis

Data are presented as mean ± SD. Data among groups were compared with one-way analysis of variance (ANOVA). A *p*-value of < 0.05 was considered significant and a *p*-value of < 0.01 was considered highly significant.

## Results

### Infection with a high-virulence strain of *H. parasuis* activated the Wnt/β-catenin signaling pathway *in vivo*

**Figure 1:**
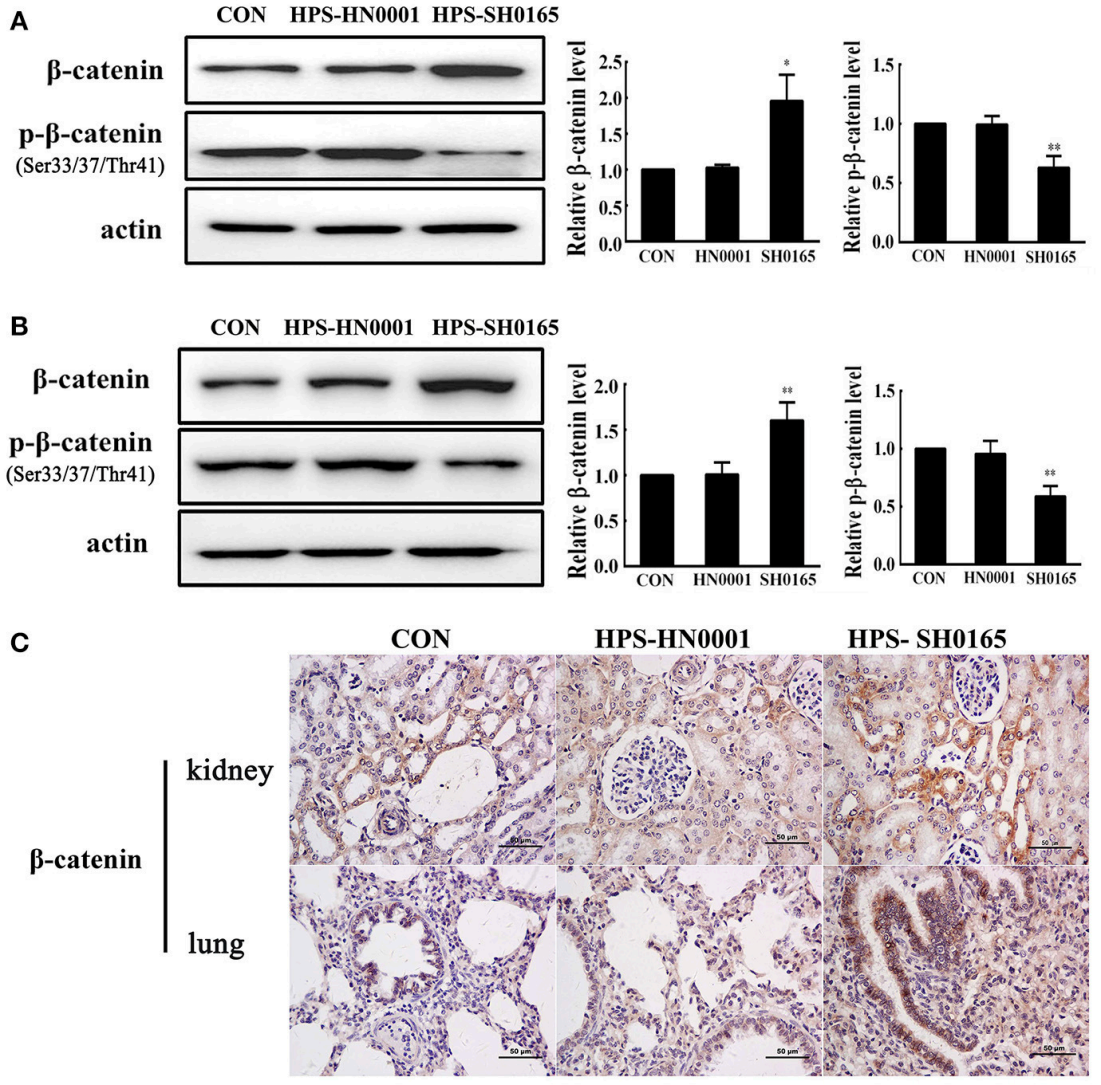
The high-virulence strain SH0165 *H. parasuis* but not the non-virulent strain HN0001 activated the Wnt/β-catenin signaling pathway *in vivo*. **(A,B)** Tissues of lung **(A)** or kidney **(B)** isolated from piglets at 24 h post injection of 2 × 10^9^ CFU *H. parasuis* or normal saline were used to analyze the expression of β-catenin and phospho-β-catenin (Ser33/37/Thr41) by Western blot. β-actin was used as the loading control. Relative β-catenin levels and p-β-catenin levels were calculated using Image J software and normalized to β-actin. Values are presented as the ratio of the infected group to the normal saline group. Representative results are shown as mean +/− SD (*n* = 3 animals). ^*^p < 0.05, ^**^p < 0.01 vs. the normal saline group. **(C)** IHC staining of the lung or kidney tissues collected from mock- or *H. parasuis*-infected piglets. β-catenin was positively stained with diaminobenzidine, and nuclei were counterstained with hematoxylin. Scale bar = 50 μm. These results are representative of lungs or kidneys of three piglets injected with either *H. parasuis* or normal saline control.

To investigate the role of the Wnt/β-catenin signaling pathway *in vivo* after *H. parasuis* infection, we examined the expression of β-catenin in kidney and lung tissues of pigs following infection with the high-virulence *H. parasuis* strain SH0165 and the non-virulent strain *H. parasuis* HN0001. As shown in Figures 1A,B, the protein level of β-catenin in SH0165-infected kidney and lung tissues was remarkably higher than that of the control and non-virulent strain HN0001 groups, while the expression of p-β-catenin in SH0165 infected kidney and lung tissues was lower than that of the control and HN0001 groups, thus indicating activation of the Wnt/β-catenin signaling pathway. Furthermore, as shown in Figure 1C, immunohistochemistry (IHC) of β-catenin demonstrated a weak constitutive expression of β-catenin in renal epithelial cells of the cortex in control and HN0001 infected samples, while showing diffuse and intense expression in SH0165 infected kidney samples. In a similar way, lung samples in the control or HN0001 infected groups also demonstrated weak β-catenin constitutive expression in lung bronchium and alveolar epithelial cells. In contrast, β-catenin exhibited much more intense expression in alveolar epithelial cells infected by SH1065. These results indicated that *H. parasuis* infection with the high-virulence strain, but not the non-virulent strain, activated the Wnt/β-catenin signaling pathway *in vivo*.

### The high-virulence strain *H. parasuis* infection activated the Wnt/β-catenin signaling pathway in PK-15 and NPTr cells

**Figure 2:**
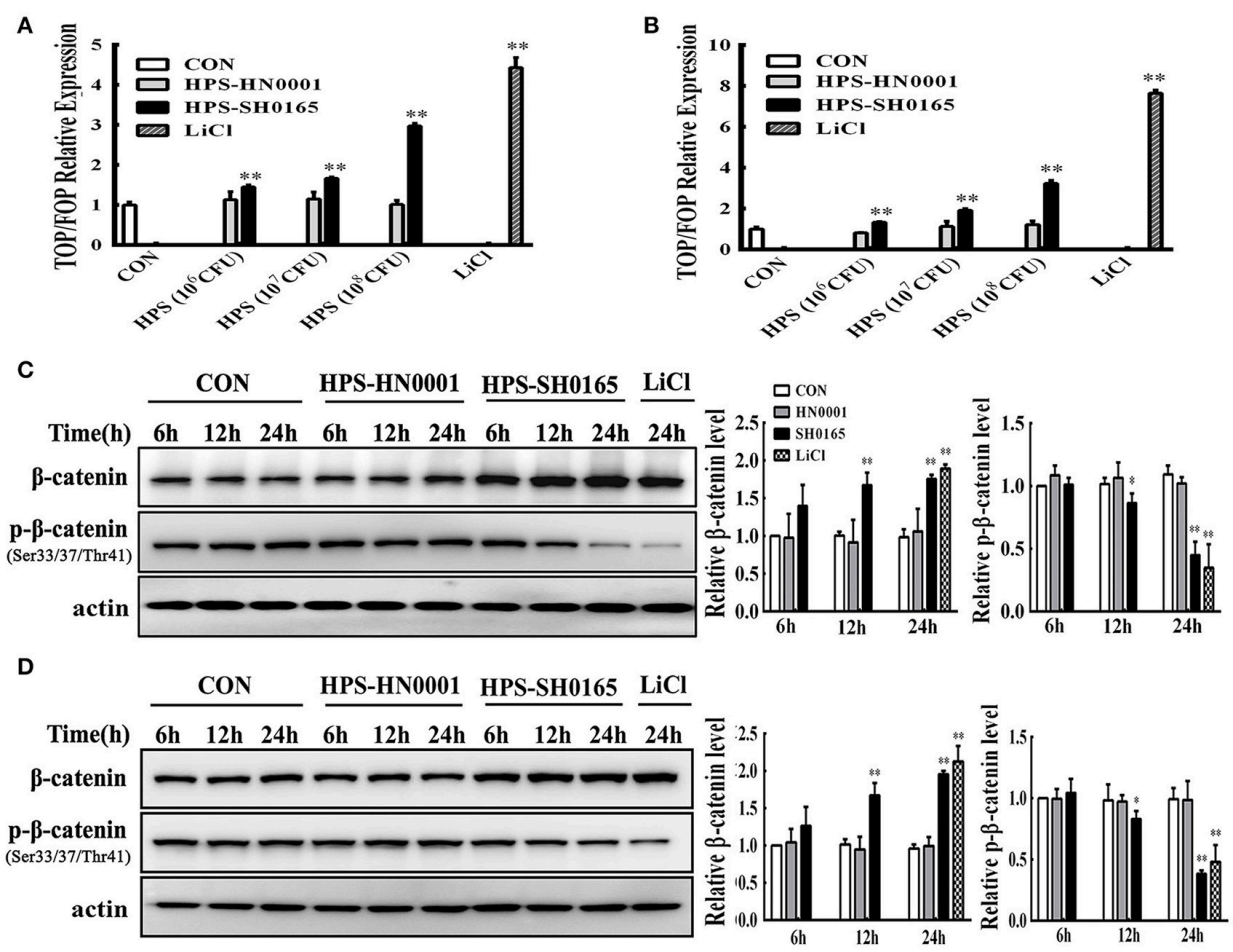
The high-virulence *H. parasuis* infection activated the Wnt/β-catenin signaling pathway in PK-15 and NPTr cells. **(A,B)** PK-15 cells **(A)** and NPTr cells **(B)** transfected with TOPflash or FOPflash were stimulated or unstimulated with *H. parasuis* strains (10^6^, 10^7^, or 10^8^ CFU/mL) or LiCl (20 mM) for 24 h. Cells were lysed and TCF transcriptional activity was determined by TOPflash/FOPflash ratio after normalization with Renilla. Representative results of three independent experiments are shown as the mean +/− SD (*n* = 3). ^**^p < 0.01 compared with uninfected group. **(C,D)** For Western blot analysis, lysates were collected from PK-15 cells **(C)** or NPTr cells **(D)** stimulated or unstimulated with *H. parasuis* (10^8^ CFU/mL) or LiCl (20 mM) for indicated periods of time and were subsequently analyzed for the expression of β-catenin and p-β-catenin (Ser33/37/Thr41). β-actin served as a loading control. Relative β-catenin levels and p-β-catenin levels were calculated using Image J software and normalized to β-actin. Values are presented as the ratio of each sample to the 6 h uninfected control (set to 1.0). Representative results of three independent experiments are shown as the mean +/− SD (*n* = 3). ^*^p < 0.05 and ^**^p < 0.01 vs. uninfected control.

Firstly, we identified that infection with the high-virulence *H. parasuis* strain could activate the Wnt/β-catenin signaling pathway *in vivo* as described above. Next, we evaluated the effects of Wnt/β-catenin signaling in both PK-15 cells and NPTr cells during *H. parasuis* infection. LiCl is an activator of Wnt signaling that inhibits the activity of GSK3β, thus stabilizing β-catenin and activating a downstream signal for nuclear translocation (Stambolic et al., 1996). TOP/FOP relative expression in SH0165-infected cells was clearly dose-dependent as demonstrated by the data in Figures 2A,B, suggesting that SH0165 infection activated the TCF/LEF transcription complex. Stimulation with LiCl markedly increased luciferase expression compared to the uninfected-control, while HN0001 infection failed to induce luciferase activity, showing that the virulence factors of the virulent strain SH0165 may play a vital role in Wnt/β-catenin activation (Figures 2A,B). As shown in Figures 2C,D, β-catenin phosphorylation was reduced while β-catenin was increased in LiCl- and SH0165- treated cells compared to control groups; however, HN0001 infection produced similar results to the control group in both PK-15 and NPTr cells. The results were consistent with those in the *in vivo* experiment.

### The high-virulence strain *H. parasuis* infection induced nuclear translocation of β-catenin in PK-15 and NPTr cells

**Figure 3:**
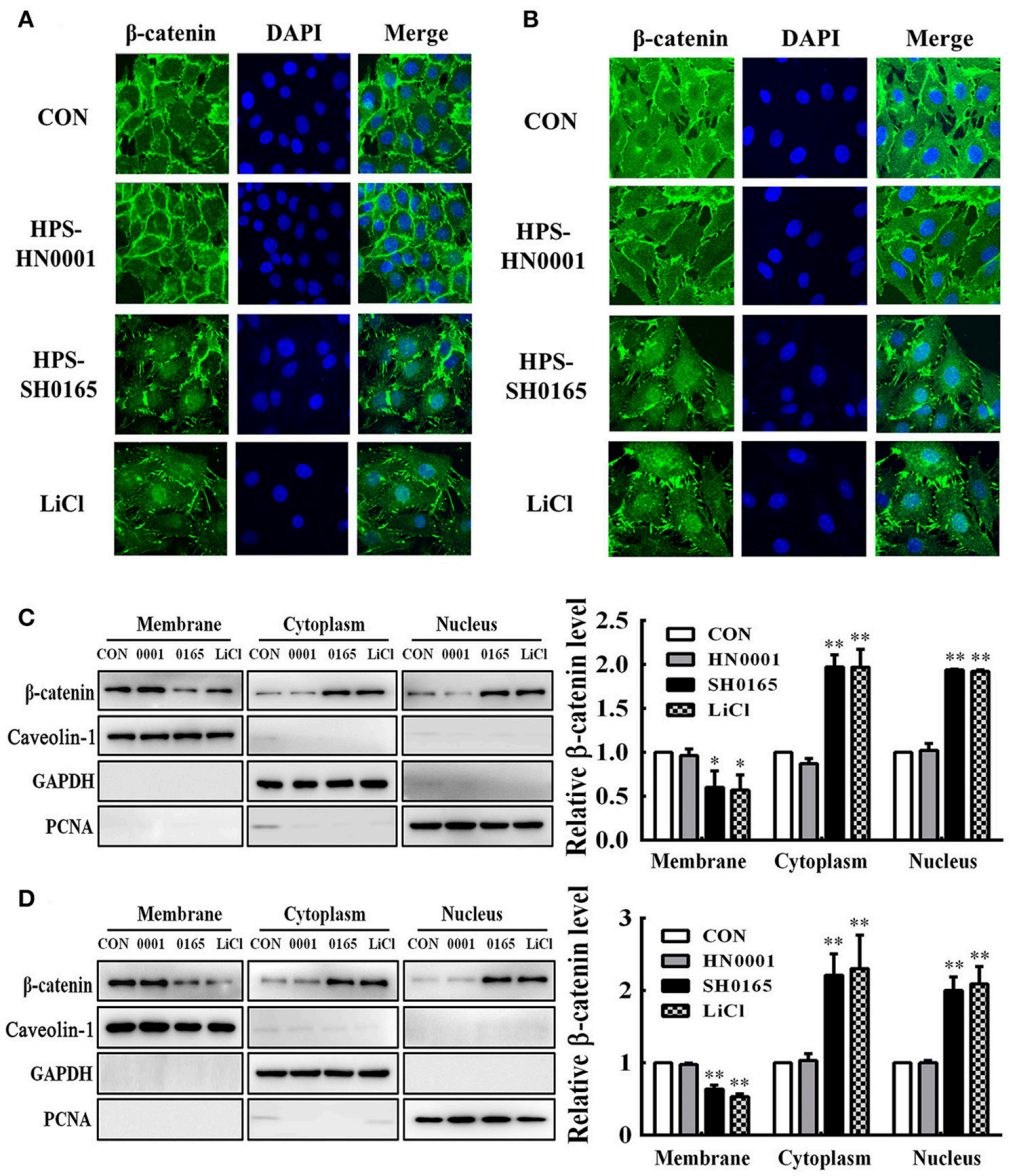
The high-virulence *H. parasuis* infection induced β-catenin to translocate into nuclear in PK-15 and NPTr cells. **(A,B)** PK-15 cells **(A)** or NPTr cells **(B)** were seeded at a density of 5 × 10^4^ on coverslips in 24-well plates. After culturing overnight, cells were infected or uninfected with SH0165 or HN0001 for 12 h. LiCl was used as the positive control. Cells were stained with anti-β-catenin antibody and then visualized with AlexaFluor 488-conjugated anti-rabbit IgG. Nuclei were stained with DAPI. **(C,D)** Protein fractions of membrane, cytoplasm, and nuclei were isolated and enriched from PK-15 cells **(C)** or NPTr cells **(D)** at 24 h after being stimulated or unstimulated with *H. parasuis* (10^8^ CFU/mL) or LiCl (20 mM), and subsequently analyzed for β-catenin expression by Western blot. Caveolin-1, GAPDH, and PCNA were used as loading controls for the membrane, cytoplasmic, and nuclear fractions, respectively. Relative β-catenin levels were calculated using Image J software and normalized to β-actin. Values are presented as the ratio of each sample to the untreated control (set to 1.0). Representative results of three independent experiments are shown as the mean +/− SD (*n* = 3). ^*^p < 0.05; ^**^p < 0.01 compared with the uninfected control.

To further investigate whether *H. parasuis* infection could activate the Wnt/β-catenin signaling pathway, the subcellular distribution of β-catenin in *H. parasuis*-infected PK-15 and NPTr cells was investigated using immunofluorescence. Intense β-catenin staining was observed in the nuclei of SH0165-infected cells and LiCl-treated cells, suggesting that stimulated β-catenin was translocated into the nucleus; in contrast, uninfected and HN0001-infected cells displayed a typical diffuse membranous/cytoplasmic staining of β-catenin (Figures 3A,B).

Using Western blotting, we subsequently assessed β-catenin subcellular dynamics during *H. parasuis* infection. Protein fractions isolated from membranes, cytoplasm, and nuclei in *H. parasuis*-infected and uninfected cells were prepared for differentially detecting the expression of β-catenin in subcellular compartments. The expression of those proteins, which include proliferating cell nuclear antigen (PCNA), glyceraldehyde 3-phosphate dehydrogenase (GAPDH), and caveolin-1 were, respectively, used as markers for nuclear (Waseem and Lane, 1990), cytoplasmic (Corsten et al., 2006), and membranous cell fractions (Chu et al., 2007). As shown in Figure 3C, D, the levels of β-catenin were reduced in membrane fractions, while they were enhanced in cytoplasmic and nuclear fractions in SH0165-infected or LiCl-treated cells. These results indicated that β-catenin was released from the connection with the proximal C-terminal domain of E-cadherin in cell membranes, accumulated in the cytoplasm, and was translocated into nuclei through SH0165 infection.

### The high-virulent strain *H. parasuis* infection induced E-cadherin degradation in PK-15 and NPTr cells

**Figure 4:**
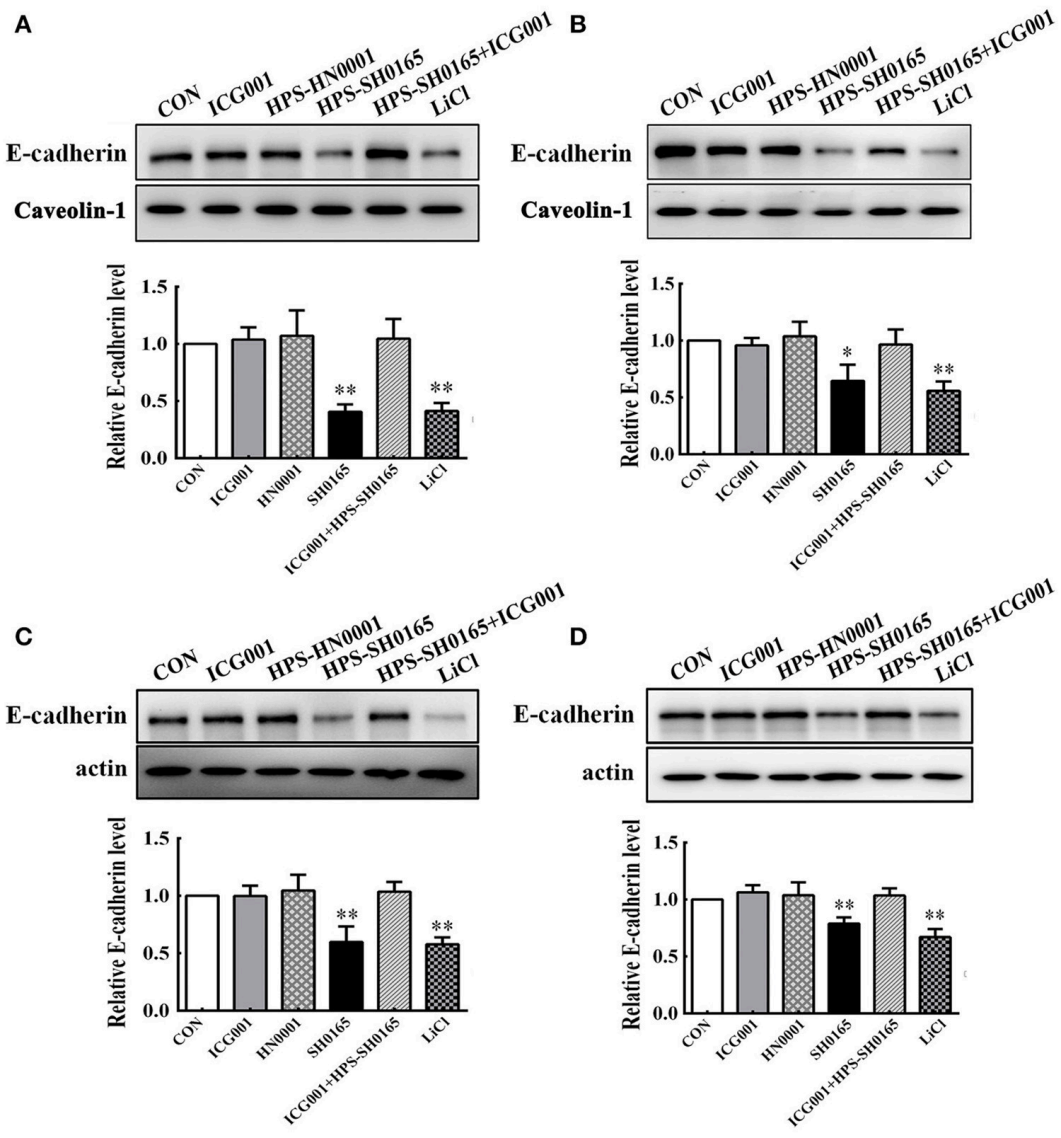
The high-virulence *H. parasuis* infection degraded E-cadherin in PK-15 and NPTr cells. Membrane protein fraction **(A,B)** and whole cell lysates **(C,D)** were isolated and enriched from PK-15 cells **(A,C)** or NPTr cells **(B,D)** treated or untreated with inhibitor ICG001 (10 μM) for 2 h before being infected or uninfected with HN0001 or SH0165 (10^8^ CFU/mL) for 24 h. E-cadherin expression levels were analyzed by Western blot. Caveolin-1 and β-actin served as the loading controls, respectively. Relative E-cadherin levels were calculated using Image J software and normalized to the loading control. Values are presented as the ratio of each sample to the control group (set to 1.0). Representative results of three independent experiments are shown as the mean +/− SD (*n* = 3). ^*^p < 0.05; ^**^p < 0.01 compared with the untreated control.

Based on the important role of the E-cadherin/β-catenin complex in forming the epithelial barrier and activating the Wnt-signaling pathway (Tian et al., 2011), we further investigated whether *H. parasuis* infection could affect the level of E-cadherin in PK-15 and NPTr cells through the Wnt/β-catenin pathway. As shown in Figure 4, the expression levels of E-cadherin protein in the membrane protein fraction or whole cell lysates of epithelial cells were severely decreased with SH0165 infection and LiCl stimulation but not HN0001 infection. Since Wnt signaling was involved in down-regulating E-cadherin (Jamora et al., 2003; ten Berge et al., 2008; Tian et al., 2011), we detected the expression of E-cadherin with the Wnt/β-catenin inhibitor ICG001, which antagonizes Wnt/β-catenin/TCF-mediated transcription and specifically binds to CREB-binding protein (CBP). We found that treatment with ICG001 blocked the degradation of E-cadherin to a certain degree in both membrane protein fractions and whole cell lysates of epithelial cells infected by *H. parasuis* (Figure 4).

### The high-virulent strain *H. parasuis* infection disrupted the interaction between β-catenin and E-cadherin, and induced monolayer leakage in PK-15 and NPTr cells

**Figure 5:**
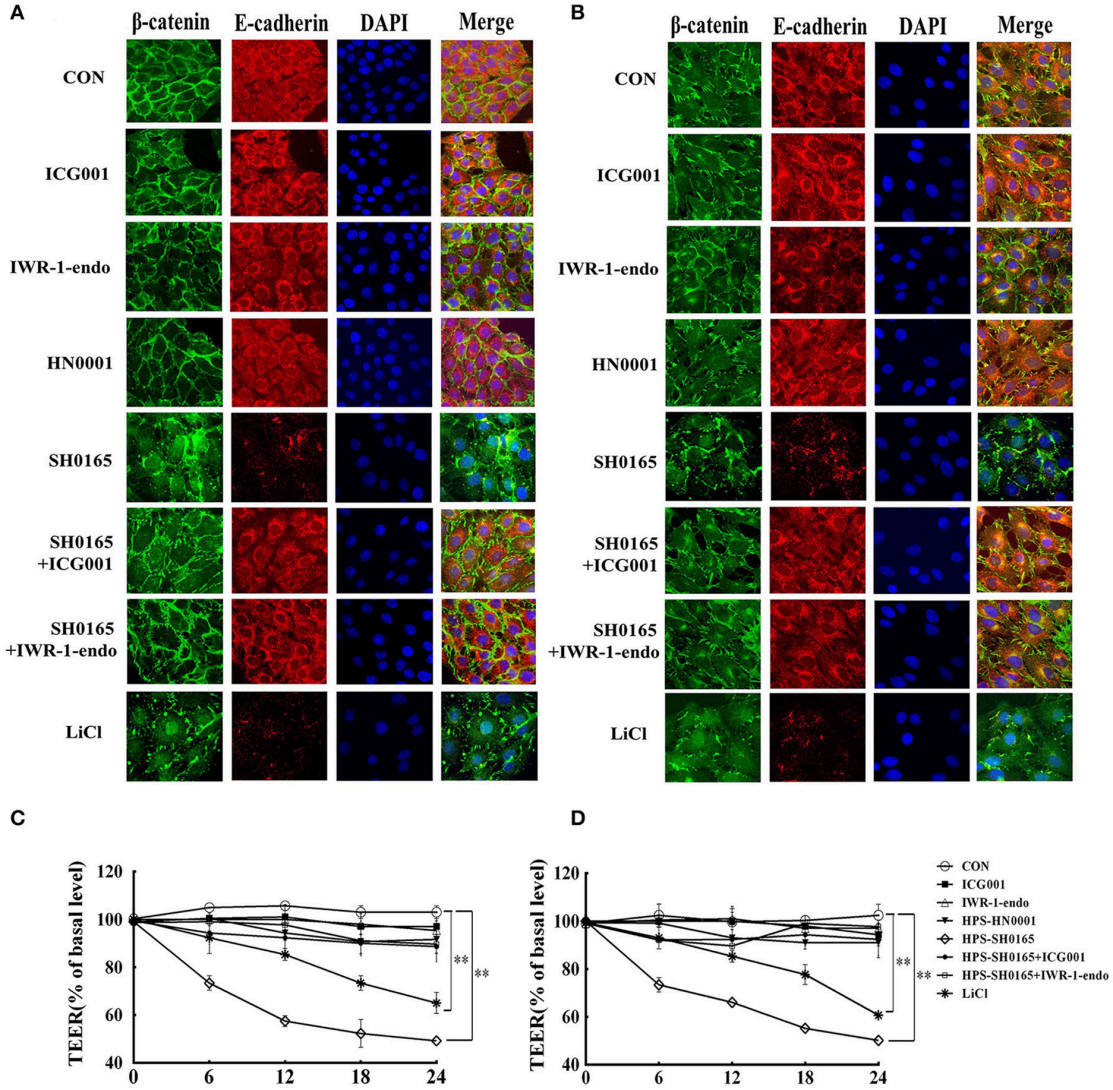
The high-virulence *H. parasuis* infection disrupted the interaction between β-catenin and E-cadherin, and induced monolayer leakage in PK-15 and NPTr cells. **(A,B)** PK-15 cells **(A)** or NPTr cells **(B)** were seeded at a density of 5 × 10^4^ on coverslips in a 24-well plate. After culturing overnight, cells were treated or untreated with 10 μM of inhibitor (ICG001 or IWR-1-endo) for 2 h before being infected or uninfected with HN0001 or SH0165 (10^8^ CFU/mL) for 12 h. Cells were stained with anti-β-catenin polyclonal antibody and anti-E-cadherin monoclonal antibody and then visualized with AlexaFluor 488-conjugated anti-rabbit IgG and AlexaFluor 555-conjugated anti-mouse IgG. Nuclei were stained with DAPI. TEER of PK-15 cells **(C)** or NPTr cells **(D)** was measured at 0, 6, 12, 18, and 24 h post-infection by *H. parasuis* (10^8^ CFU/mL) or LiCl (20 mM). Inhibitors (ICG001 and IWR-1-endo) were added to upper compartments 2 h before infection. TEER levels were calculated and presented as a percentage of pretreatment TEER. Representative results of three independent experiments are shown as the mean +/− SD(*n* = 3). ^**^p < 0.01 compared with uninfected group.

Considering that β-catenin/E-cadherin based anchoring junctions are important for AJs, which in turn maintain epithelial integrity (Tian et al., 2011), we evaluated the effect of *H. parasuis* infection on epithelial integrity through β-catenin signaling. In this experiment, confocal images revealed that E-cadherin protein formed a regular distribution pattern in uninfected and HN0001-infected cells, while it was distributed chaotically and degraded 12 h after SH0165 infection or LiCl stimulation (Figures 5A,B). Furthermore, SH0165 infection but not HN0001 led to a decrease in co-localization of β-catenin and E-cadherin (especially in E-cadherin), and thus disrupted their conjunction in both PK-15 and NPTr cells compared with the control (Figures 5A,B). To detect the change of epithelial integrity in PK-15 and NPTr cells, transepithelial resistance (TEER) was measured at 0, 6, 12, 18, and 24 h post-infection with *H. parasuis* (10^8^ CFU/mL) or LiCl (20 mM). As shown in Figures 5C,D, the levels of transepithelial resistance in PK-15 and NPTr cells were significantly reduced by 50 to 60% in the SH0165 but not HN0001 infected groups at 24 h post-infection. Of greater interest, treatment with Wnt/β-catenin inhibitor ICG001 and IWR-1-endo partly restored the interaction between E-cadherin and β-catenin as well as the barrier defect wrecked by SH0165 to a certain degree, suggesting an important role of Wnt/β-catenin signaling for maintaining the integrity of epithelial cells during *H. parasuis* infection.

### The high-virulent strain *H. parasuis* infection initialized EMT process dependent on Wnt/β-catenin signaling pathway in PK-15 and NPTr cells

**Figure 6:**
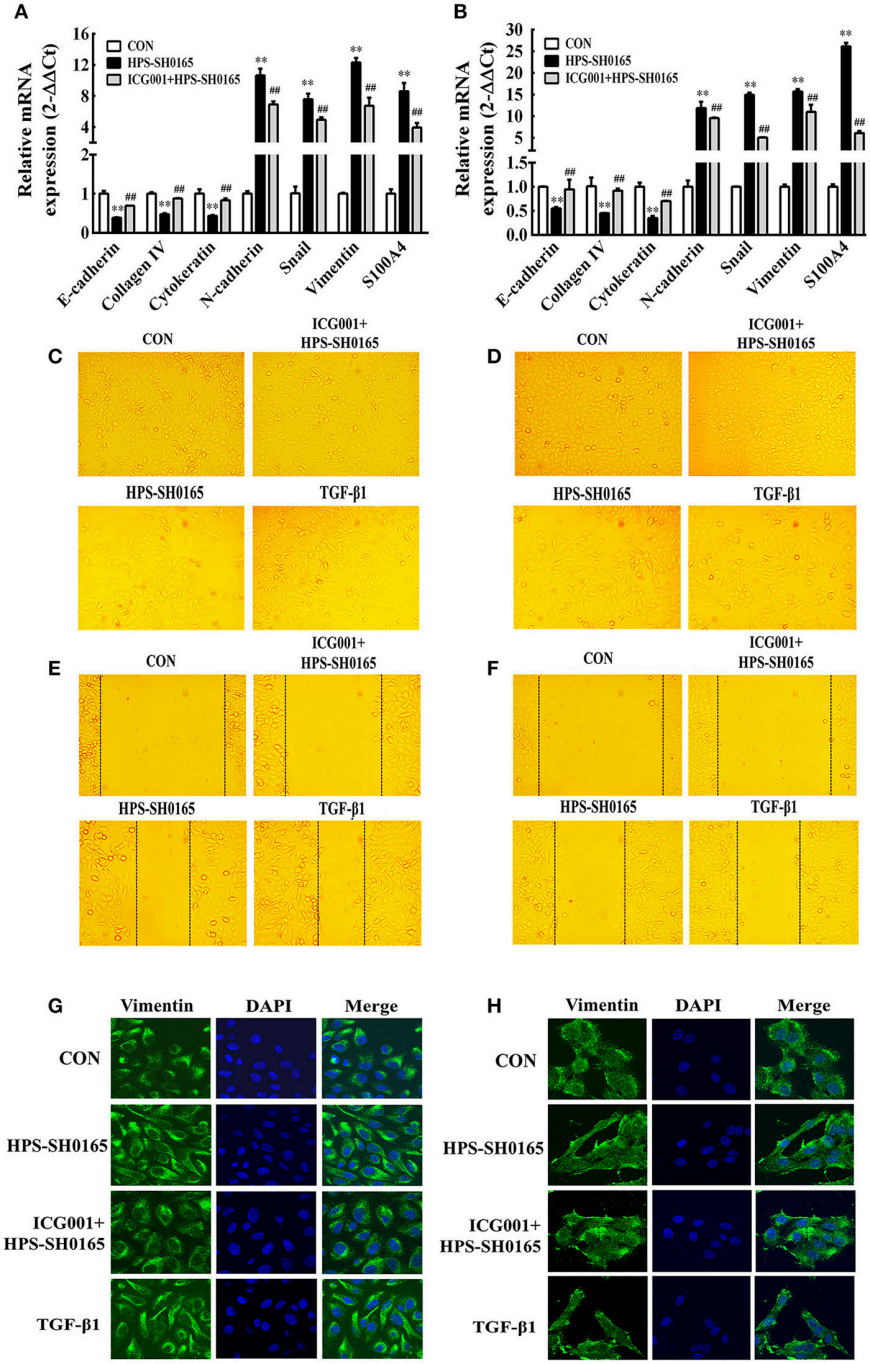
The high-virulent strain *H. parasuis* infection initialized EMT process dependent on Wnt/β-catenin signaling pathway in PK-15 and NPTr cells. **(A,B)** The mRNA expression level of EMT characteristic genes in PK-15 cells **(A)** and NPTr cells **(B)** was analysis by qRT-PCR at 12 h after infected with SH0165 (10^8^ CFU/mL). Cells were treated or untreated with ICG001 for 2 h before infection. The relative mRNA levels of each sample were normalized to corresponding GAPDH mRNA levels. Representative results of three independent experiments are shown as the mean +/− SD (*n* = 3). ^**^p < 0.01 compared with the uninfected control. **##***p* < 0.01 compared with the SH0165 infected group **(C–F)** PK-15 cells **(C,E)** and NPTr cells **(D,F)** were treated or untreated with ICG001 for 2 h before stimulated or unstimulated with SH0165 (10^8^ CFU/mL) for 12 h. TGF-β1 (10 ng/mL) was used as the positive control. The cellular morphology **(C,D)** was obtained by microscopy and the wound healing assay **(E,F)** was used to detect cell migration. **(G,H)** PK-15 cells **(G)** or NPTr cells **(H)** were seeded at a density of 5 × 10^4^ on coverslips in 24-well plates. After culturing overnight, cells were treated or untreated with ICG001 (10 μM) for 2 h before infected or uninfected with SH0165 (10^8^ CFU/mL) for 12 h. TGF-β1 (10 ng/mL) was used as the positive control. Cells were stained with anti-Vimentin monoclonal antibody and then visualized with AlexaFluor 488-conjugated anti-rabbit IgG. Nuclei were stained with DAPI.

To further investigate the functional effects of Wnt/β-catenin signaling pathway activated by the *H. parasuis* infection, we explored the possibility for initiation of EMT process, in which the E-cadherin repression is a fundamental event (Serrano-Gomez et al., 2016). Real-time RT-PCR revealed the characteristic gene-expression changes after *H. parasuis* infection. The results showed a significantly suppression of epithelial marker genes E-cadherin, collagen IV and cytokeratin, as well as increased expression of mesenchymal marker genes N-cadherin, snail, vimentin and S100A4 at 12 h after infection with SH0165 (10^8^ CFU/mL) (Figures 6A,B). In Figures 6C,D, PK-15 and NPTr cells changed from the epithelial-like form to a spindle-like shape, which is feature of mesenchymal phenotype (Kühn et al., 2015), after 12 h treatment of SH0165 (10^8^ CFU/mL) or TGF-β1 (10 ng/mL). The morphological change was further demonstrated by confocal (Figures 6G,H), which showed the spindle-shaped distribution of vimentin after infection of SH0165 (10^8^ CFU/mL) or TGF-β1 (10 ng/mL). This result is accordance with previous reports depicting the rearrangement of vimentin during EMT process (Alcaraz et al., 2013). Furthermore, the migration ability of PK-15 and NPTr cells was measured by wound healing assay, and the results showed that treatment with SH0165 (10^8^ CFU/mL) or TGF-β1 (10 ng/mL) substantially increased the cellular migration compared with the untreated group (Figures 6E,F). These results proved that infection of high-virulent *H. parasuis* strain might initialize EMT process in PK-15 and NPTr cells. Moreover, treatment of Wnt/β-catenin inhibitor ICG001 could markedly recovered the morphological change, migration ability as well as the characteristic gene-expression changes of EMT, indicating that Wnt/β-catenin signaling pathway may play an important role in regulating the EMT process induced by *H. parasuis*.

### Knockdown of Wnt target genes significantly recover the EMT process induced by the high-virulent strain *H. parasuis*

**Figure 7:**
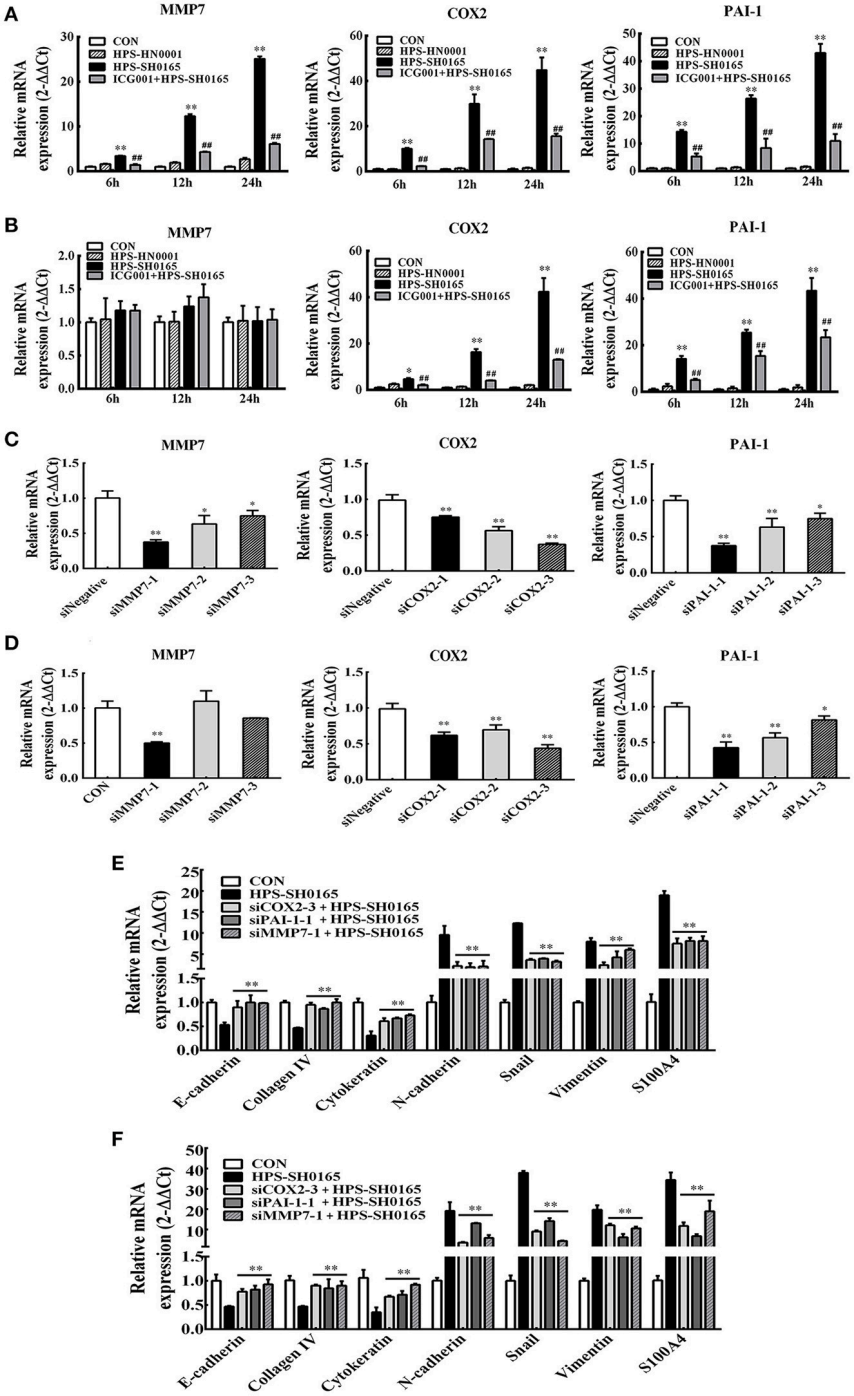
The high-virulent strain H. parasuis infection initialized EMT process dependent on Wnt/β-catenin signaling pathway in PK-15 and NPTr cells. **(A,B)** MMP7, COX2, and PAI-1 mRNA expression in *H. parasuis* -infected (10^8^ CFU/mL) PK-15 cells **(A)** and NPTr cells **(B)** for indicated periods of time was analyzed by qRT-PCR. Cells were treated or untreated with ICG001 for 2 h before infection. The relative mRNA levels of each sample were normalized to corresponding GAPDH mRNA levels. Representative results of three independent experiments are shown as the mean +/− SD (n = 3). ^*^p < 0.05; ^**^p < 0.01 compared with the uninfected control. **##***p* < 0.01 compared with the SH0165 infected group **(C,D)** PK-15 cells **(C)** and NPTr cells **(D)** seeded at 24 well plates were transfected with 15 pmol/well sequence-specific siRNAs or siNegative by 1.5 μg/well lipofectamine 2000. Twenty Four hour after transfection, cells were collected to detect the expression level of endogenous MMP7, COX2, PAI-1. **(E,F)** The expression level of EMT characteristic genes was detected by qRT-PCR. PK-15 cells **(E)** and NPTr cells **(F)** seeded at 24 well plates were transfected with 15 pmol/well sequence-specific siRNAs. Cells of mock group and SH0165-infected group were transfected with 15 pmol/well siNegative. Twenty-Four after transfection, cells were infected with SH0165 (10^8^ CFU/mL) for 12 h and collected for qRT-PCR analysis. The relative mRNA levels of each sample were normalized to corresponding GAPDH mRNA levels. Representative results of three independent experiments are shown as the mean +/− SD (*n* = 3). ^**^p < 0.01 compared with the SH0165 infected group.

To further explore the role of Wnt-signaling pathway in regulating the EMT process induced by *H. parasuis*, we evaluated the function of three Wnt target genes, MMP7, COX2 and PAI-1, which have been proved to be associated with cell structural change and the process of EMT in some tumor cells (Neil et al., 2008; Omori et al., 2016; Zhang et al., 2017). Firstly, we detected the expression of MMP7, COX2, and PAI-1 genes after infection of *H. parasuis*, as shown in Figures 7A,B, real-time RT-PCR showed SH0165 infection but not HN0001 led to a significant upregulation of expression of all three genes in PK-15 cells (Figure 7A) and only expression of COX2 and PAI-1 in NPTr cells (Figure 7B) compared to uninfected cells. Moreover, the increased expression could be significantly inhibited after treated with ICG001. To further investigate the role of three target genes in EMT process induced by *H. parasuis*, three double-stranded siRNAs of each gene were designed (Table 2). The most effectively siRNAs were chosen for subsequent experiments based on the results of real-time RT-PCR (Figures 7C,D). As a result, siMMP7-1, siCOX2-3, and siPAI-1-1 were chosen to subsequently transfected into PK-15 and NPTr cells to detect the characteristic gene-expression of EMT. As shown in Figures 7E,F, both suppression of epithelial marker genes and increased expression of mesenchymal marker genes were all markedly recovered after the knockdown of any one of three target genes. These results indicated that Wnt/β-catenin signaling pathway may regulate the EMT process induced by *H. parasuis* through the Wnt target genes.

## Discussion

In the present study, animal experimentation confirmed that the Wnt/β-catenin signaling pathway was activated with a decreased phosphorylation level of β-catenin (Ser33/37/Thr41) and an increased accumulation of β-catenin in lung and kidney tissues of pigs infected with a high-virulence strain of *H. parasuis* (Figure 1). Consistent with the results *in vivo*, infection of epithelial cells with *H. parasuis* caused an increased accumulation of β-catenin, leading to an up-regulation of dose-dependent Wnt/β-catenin transcriptional activity (Figure 2). Increased accumulation of β-catenin is also observed in nonpathogenic *Salmonella typhimurium* and the human pathogen *Helicobacter pylori* (Silva-Garcia et al., 2014). AvrA, a bacterial effector in *Salmonella*, inhibits the ubiquitination of β-catenin in human epithelia, resulting in the accumulation and nuclear translocation of β-catenin (Sun et al., 2004). *Helicobacter pylori*-induced activation of the canonical Wnt/β-catenin signaling pathway in gastric epithelial cells depends on VirB7 (required for a functional type 4 secretion system) (Wessler and Backert, 2008; Gnad et al., 2010). It is also worth mentioning that both AvrA and VirB7 are not homologous with any protein in *H. parasuis* SH0165, suggesting that different bacteria employ individual mechanisms to regulate the Wnt/β-catenin signaling pathway. In contrast, β-catenin protein expression is decreased *in vivo* and *in vitro* during infection with *Pseudomonas aeruginosa* (Chen et al., 2013) and Wnt/β-catenin signaling is significantly reduced by aerosol infection of mice with *Mycobacterium tuberculosis* (Neumann et al., 2010). These apparently contradictory results were obtained when animals or cells were stimulated with different pathogenic bacteria, suggesting that the role of Wnt/β-catenin may depend on the stimulus, the cell type, the activation context, and its crosstalk with other signaling pathways (Silva-Garcia et al., 2014).

Our results showed that Wnt/β-catenin pathway activated by *H. parasuis* infection brought epithelial barrier disruption with a reduction in E-cadherin. However, we could not determine the exact role of Wnt/β-catenin signaling in regulating the expression of E-cadherin. E-cadherin could be down-regulated by inhibiting E-cadherin expression or cleavage/degradation. The IWR-1-endo is an upstream inhibitor of the Wnt pathway, and its inhibitory effect may be associated with (i) the so-called destruction complex consisting of GSK3β, Axin, and APC, (ii) accumulation of β-catenin in space, (iii) nuclear β-catenin/LEF mediated gene transcription, while ICG001 only targets the latter. Not surprisingly, treatment with inhibitor ICG001 or IWR-1-endo remarkably depressed the reduction of E-cadherin induced by *H. parasuis* infection almost to the same level, suggesting that this was due to nuclear β-catenin/LEF mediated suppression of E-cadherin expression (Figure 5). This outcome is reasonable since Wnt/β-catenin signaling has been found to directly control the expression of E-cadherin in skin keratinocytes; furthermore, many Wnt targeted transcription factors, such as Snail, and Slug, inhibit the E-cadherin gene promoter and influence cadherin adhesion (Jamora et al., 2003; Heuberger and Birchmeier, 2010). It was reported that loss of E-cadherin not only disrupts cell-cell junction but also provokes an EMT, attended by increased cellular motility, invasiveness in cancer cells (Onder et al., 2008), suggesting that the *H. parasuis* infection may result in the transition of epithelial cells to EMT phenotype depending on Wnt signaling pathway.

Furthermore, we demonstrated that *H. parasuis* induced the transcription of Wnt target genes including MMP7, COX2, PAI-1 in cells, which were reported associated with cell structural change of the cells and the process of EMT. For example, the cleavage of E-cadherin by MMP-7 mediated epithelial cell proliferation and loss of cell-cell contact (Lynch et al., 2010). In addition, MMP7 is involved in the breakdown of extracellular matrixes during disease processes, such as arthritis and cancer metastases (Ye et al., 2007; Xu et al., 2016), among which arthritis is a typical symptom caused by *H. parasuis*; COX2 has been associated with HIV Tat-induced up-regulation of tight junction protein and disruption of the integrity of the blood-brain barrier (Pu et al., 2007). PAI-1 is involved in wound healing including severe fibrotic lesions, tissue remodeling of extracellular matrix accumulation (He et al., 2010). In addition, previous studies indicated that the function of Wnt signaling target genes including MMP7, COX2, PAI-1 was all related to EMT (Neil et al., 2008; Omori et al., 2016; Zhang et al., 2017). In this study, the interference of MMP7, COX2, PAI-1 could significantly recover repressed epithelial markers and promoted mesenchymal markers seduced by *H. parasuis* infection (Figure 7) suggesting that EMT initiated by *H. parasuis* infection depended on Wnt signaling pathway in epithelial cell.

Evidences including the expression changes of selected EMT markers, morphological changes and increased migratory capabilities from this study (Figure 6) confirmed the existence of EMT during *H. parasuis* infection. It was found that the mRNA expression of typical EMT transcription factor Snail was markedly up-regulated during *H. parasuis* infection depending on Wnt signaling pathway (Figure 6), E-cadherin, the downstream targets of Snail (Yook et al., 2005), showed significantly suppression in the mRNA level (Figure 6), and canonical EMT biomarker Vimentin, another target of Snail was up-regulated and rearranged to a spindle-like shape in epithelial cells (Figure 6). All those changes promoted epithelial cells lose their adhesions and apical–basal polarity and reorganize their cytoskeleton toward a mesenchymal phenotype (Medici et al., 2008). In all, EMT of the epithelial cells in organs contributes to tissue repair by generating fibroblasts, but it also endows cells with migratory and invasive properties and adversely causes organ fibrosis through a variety of mechanisms (Thiery et al., 2009). This kind of EMT termed as Type II EMT usually occurs following tissue injuries such as inflammation (Yu et al., 2018). Previous observations revealed the important role of Type II EMT during chronic inflammation associated diseases leading to tissue fibrosis (Rout-Pitt et al., 2018), but seldom in acute inflammation. It was also reported that the process of EMT of tubular epithelial cells taking place in acute kidney injury was entirely consistent with what happened in chronic kidney injury, but the outcome was completely different, regeneration or fibrosis formation, depending on a different microenvironment or the duration of the injury (Jiang et al., 2013).

In conclusion, *H. parasuis* infection activated the canonical Wnt/β-catenin signaling pathway leading to the disruption of adherens junctions with a sharp degradation of E-cadherin as well as an increase in the permeability of epithelial cells, and initialized EMT process dependent on Wnt/β-catenin signaling pathway in epithelial cells, which involved the expression changes of selected EMT markers, morphological changes and increased migratory capabilities. All these phenotype changes depending on Wnt signaling pathway contributed to disrupting epithelial barriers, altering cell structure and increasing cell migration, which may result in severe acute systemic infection characterized by fibrinous polyserositis during *H. parasuis* infection.

## Author contributions

HJ and KH designed the research. RL, HJ, HZ, RZ, and DB conducted the experiments. KH, YL, RH, and YC acquired the data. HJ, RL, KH, and YL analyzed the data. XH and RZ provided the essential materials. HJ, HZ, and KH wrote the manuscript.

### Conflict of interest statement

The authors declare that the research was conducted in the absence of any commercial or financial relationships that could be construed as a potential conflict of interest.

## Supplementary material

Supplemental Figure 1 Quantity of *H. parasuis* SH0165 at indicated time after adding into cells. The Wnt inhibitor ICG001 and IWR-1-endo was added into cell culture medium 2 h before *H. parasuis* infection. At 0, 6, 12, and 24 h after infected with *H. parasuis* SH0165, 100 μL culture medium of PK-15 **(A)** and NPTr **(B)** cell samples was taken out to test the quantity of bacteria.
