# Supplementary figures and images for: Haemophilus parasuis Infection Disrupts Adherens Junctions and Initializes EMT Dependent on Canonical Wnt/β-Catenin Signaling Pathway
Source: Front Cell Infect Microbiol. 2018 Sep 12;8:324. doi: 10.3389/fcimb.2018.00324 (PMC6143654; doi:10.3389/fcimb.2018.00324)

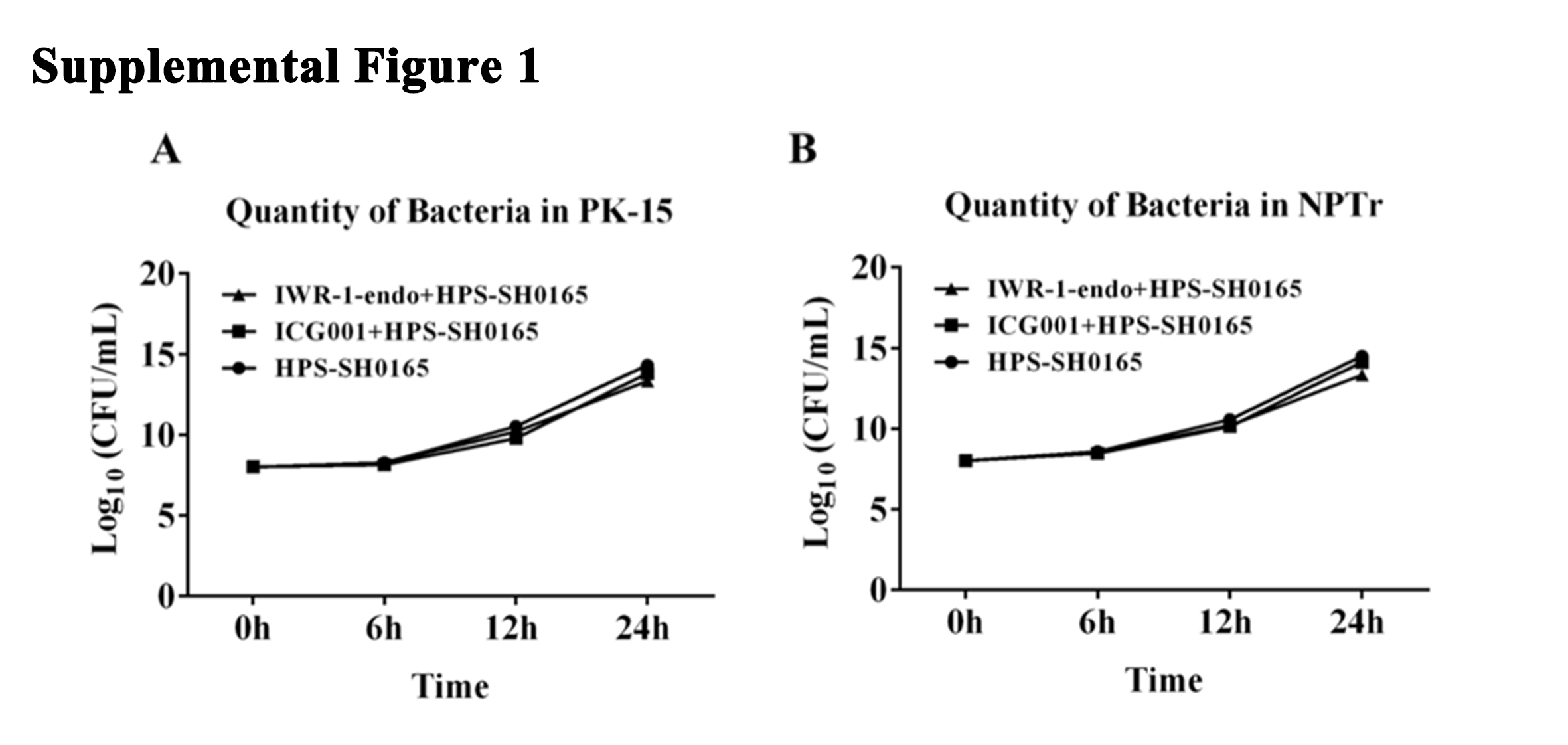

Supplement: Supplemental Figure 1 — Quantity of H. parasuis SH0165 at indicated time after adding into cells. The Wnt inhibitor ICG001 and IWR-1-endo was added into cell culture medium 2 h before H. parasuis infection. At 0, 6, 12, and 24 h after infected with H. parasuis SH0165, 100 μL culture medium of PK-15 (A) and NPTr (B) cell samples was taken out to test the quantity of bacteria. [file Image_1.TIF]
